# Deep sequencing of hepatitis C virus hypervariable region 1 reveals no correlation between genetic heterogeneity and antiviral treatment outcome

**DOI:** 10.1186/1471-2334-14-389

**Published:** 2014-07-13

**Authors:** Kamila Caraballo Cortés, Osvaldo Zagordi, Karol Perlejewski, Tomasz Laskus, Krzysztof Maroszek, Iwona Bukowska-Ośko, Agnieszka Pawełczyk, Rafał Płoski, Hanna Berak, Andrzej Horban, Marek Radkowski

**Affiliations:** 1 Department of Immunopathology of Infectious and Parasitic Diseases, Medical University of Warsaw, 3c Pawińskiego Street, 02-106 Warsaw, Poland; 2 Institute of Medical Virology, University of Zurich, Winterthurerstrasse, 190 8057 Zurich, Switzerland; 3 Department of Medical Genetics, Medical University of Warsaw, 3c Pawińskiego Street, 02-106 Warsaw, Poland; 4 Hospital for Infectious Diseases, 37 Wolska Street, 01-201 Warsaw, Poland; 5 Clinics of Infectious Diseases, Medical University of Warsaw, 37 Wolska Street, 01-201 Warsaw, Poland

**Keywords:** Hypervariable region 1, Ultra-deep sequencing, Treatment, Genetic heterogeneity, Hepatitis C virus, Quasispecies, Pyrosequencing

## Abstract

**Background:**

Hypervariable region 1 (HVR1) contained within envelope protein 2 (E2) gene is the most variable part of HCV genome and its translation product is a major target for the host immune response. Variability within HVR1 may facilitate evasion of the immune response and could affect treatment outcome. The aim of the study was to analyze the impact of HVR1 heterogeneity employing sensitive ultra-deep sequencing, on the outcome of PEG-IFN-α (pegylated interferon α) and ribavirin treatment.

**Methods:**

HVR1 sequences were amplified from pretreatment serum samples of 25 patients infected with genotype 1b HCV (12 responders and 13 non-responders) and were subjected to pyrosequencing (GS Junior, 454/Roche). Reads were corrected for sequencing error using ShoRAH software, while population reconstruction was done using three different minimal variant frequency cut-offs of 1%, 2% and 5%. Statistical analysis was done using Mann–Whitney and Fisher’s exact tests.

**Results:**

Complexity, Shannon entropy, nucleotide diversity per site, genetic distance and the number of genetic substitutions were not significantly different between responders and non-responders, when analyzing viral populations at any of the three frequencies (≥1%, ≥2% and ≥5%). When clonal sample was used to determine pyrosequencing error, 4% of reads were found to be incorrect and the most abundant variant was present at a frequency of 1.48%. Use of ShoRAH reduced the sequencing error to 1%, with the most abundant erroneous variant present at frequency of 0.5%.

**Conclusions:**

While deep sequencing revealed complex genetic heterogeneity of HVR1 in chronic hepatitis C patients, there was no correlation between treatment outcome and any of the analyzed quasispecies parameters.

## Background

Hepatitis C virus (HCV) circulates within the infected host as a pool of related but distinct genetic variants (quasispecies); [1]. The genetic variability is mainly generated by viral RNA-dependent RNA polymerase (RdRp) which lacks a proof-reading activity [2]. Genes encoding envelope E1 and E2 proteins, especially the hypervariable 1 region (HVR1) of E2, display the highest genetic variability within the whole HCV genome [3]. HVR1 contains sequences encoding important immune epitopes; thus genetic variability within this region may facilitate evasion of the immune responses and is largely shaped by the immune pressure of the host [4-8]. Complexity and evolution of HVR1 quasispecies was reported to be predictive factor of the outcome of natural infection [9,10].

Antiviral treatment protocols using interferon and ribavirin have limited efficacy and are plagued by side effects, which often require premature discontinuation of therapy. Factors known to be associated with treatment outcome include both host (i.e. IL28B gene polymorphisms, race, sex, age) as well as viral factors (genotype, serum load and genetic heterogeneity); [11-13].

Interferon and ribavirin treatment is based largely on direct antiviral effect as well as immunomodulation [14]. Thus, HVR1 heterogeneity could facilitate treatment failure since coexistence of multiple antigenic variants could increase the probability of positive selection of those effectively evading immune pressure induced by treatment [15,16]. However, despite attempts to correlate HVR1 heterogeneity with antiviral treatment outcome, published studies are inconclusive [17-20].

Recent years brought the advent of ultra-deep sequencing techniques which enable parallel sequencing of multiple sequences present in a sample, thus providing better insight into the quasispecies phenomenon. Pyrosequencing (454/Roche), one of the available deep sequencing platforms, is capable of reading sequences up to 1 kb, and it was used successfully for sequence analysis of human immunodeficiency virus (HIV) and HCV [21-25].

Similarly, our previous analysis of HVR1 in chronic HCV infection confirmed the utility of pyrosequencing for HCV haplotypes inference, including identification of very rare variants constituting as little as 0.1% of the whole population [26].

The present study employed pyrosequencing to explore HVR1 complexity and variability in pretreatment serum samples of patients treated with pegylated interferon α (PEG-IFN α ) and ribavirin. We demonstrated that complexity, Shannon entropy, nucleotide diversity per site, genetic distance and the number of genetic substitutions were not significantly different between responders and non-responders, when analyzing populations present at ≥1%, ≥2% and ≥5% frequency.

## Methods

### Patients

Our prospective study involved 95 chronic hepatitis C patients undergoing treatment at the Outpatient Clinic of the Hospital for Infectious Diseases in Warsaw from June 2010 to December 2012. Out of this cohort, twenty five patients were selected according to the following criteria: chronic infection with genotype 1b HCV, no previous antiviral treatment, no co-infection with HBV or HIV, no history of intravenous drugs use. In addition, patients had to achieve complete early viral response (cEVR), defined as undetectable HCV RNA in the serum after 12 weeks of treatment and, subsequently, sustained viral response (SVR) defined as undetectable HCV RNA in the serum 6 months post-treatment (responders, n = 12), or experience no viral load reduction ≥ 2 log at week 12 of treatment (non-responders, NR, n = 13). No statistically significant differences were found between responders and non-responders in mean alanine aminotransferase activity, pretreatment viral load, age, liver grading and staging or sex (Table 1). Viral load was measured by RealTime HCV assay (Abbott), sensitivity: 12 IU/mL, while qualitative evaluation was performed by COBAS Amplicor HCV Test, v2.0 (Roche Diagnostics) which has sensitivity limit of 50 IU/ml. Treatment consisted of pegylated interferon α (Pegasys, Roche), 180 μg per week, n = 13 or Pegintron (Schering-Plough) at dose 1,5 μg/kg of body weight, n = 12 and Ribavirin (Copegus, Roche), 1000 mg/day (body mass < 75 kg) or 1200 mg/day (body mass > 75 kg), n = 13 or Rebetol (Schering-Plough), 800 mg/day (body mass < 64 kg), 1000 mg/day (body mass 65–85 kg), 1200 mg (body mass 86–105 kg) or 1400 mg (body mass > 105 kg), n = 12. Responders were treated for 48 weeks, whereas in non-responders the therapy was stopped after 12 weeks. The study was approved by the Institutional Bioethical Committee (consent N^o^ KB/107/2010) and all patients provided informed consent.

**Table 1:** Clinical and virological characteristics of 25 studied patients infected with genotype 1b

	**Treatment responders SVR+, n = 12**	**Treatment non-responders SVR-, n = 13**	***P***
Complete early viral response (cEVR)	12	0	-
Age (years)^*^	42.6 ± 17.9	50.1 ± 12.4	NS
Sex (M/F)	6/6	7/6	NS
Alanine aminotransferase levels [U/l]^*^	95.9 ± 74.1	109.2 ± 50.1	NS
Liver histology^*, §^			
Grading	1.1 ± 0.4	1.1 ± 0.4	NS
Staging	1.3 ± 1.3	1.7 ± 0.8	NS
Pretreatment viral load (IU/ml)^* , **^	1.2 × 10^6^ ± 1.2 × 10^6^	1.5 × 10^6^ ± 1.2× 10^6^	NS

^*^mean ± SD.

^**^measured by RealTime HCV assay (Abbott), sensitivity: 12 IU/mL.

^§^according to the METAVIR Histologic Scoring System.

### HVR1 amplification

HVR1 amplification was performed from pretreatment serum samples as described previously [26]. In brief, viral RNA was extracted from 250 μl of serum by modified guanidinium thiocyanate-phenol/chlorophorm method, then subjected to reverse transcription at 37°C for 30 minutes using AccuScript High Fidelity Reverse Transcriptase (Agilent Technologies). A fragment of E2 region containing HVR1 was amplified in two-step PCR using FastStart High Fidelity Taq DNA Polymerase (Roche). Primers for the second round PCR contained tags recognized by GS Junior sequencing platform, standard 10-nucleotide multiplex identifiers (MID) and target-specific sequence.

### Cloned HVR1 sequence

To determine the inherent sequencing error, amplified HVR1 from one sample was purified by Wizard SV Genomic DNA Purification System (Promega) and cloned into TOPO TA vector using TOPO TA Cloning Kit (Invitrogen). Plasmid DNA was extracted from bacterial culture using Quick Plasmid Miniprep Kit (Life technologies). Subsequently, pyrosequencing-specific tags with multiplex identifier (MID) were introduced by means of PCR using plasmid sequence as a target and sample was subjected to pyrosequencing.

### Pyrosequencing

Each amplicon was purified from agarose gel by QIAquick Gel Extraction kit (Qiagen) and then by Agencourt AMPure XP beads (Beckman Coulter) using 1.6:1 ratio of beads to sample. Products were quantified by dsDNA HS Qubit® Assay Kit (Life Technologies), fourteen samples were pooled in equivalent amounts and of 3 × 10^7^ DNA copies were subjected to emulsion PCR using GS Junior Titanium emPCR Kit (Lib-A). After initial denaturation at 94°C for 1 minute, the reaction was run for 50 cycles of 94°C for 30 seconds, 58°C for 4 minutes and 30 seconds, and 68°C for 30 seconds. DNA library beads enrichment was carried out according to the emPCR Amplification Method Manual Lib-A (Roche), with the exception that the number of bead washes was 15. The required input of 500 000 enriched beads was loaded onto the Pico Titer Plate (PTP) and sequencing was carried out for 200 cycles using full processing mode for amplicons (GS Junior Sequencer, 454/Roche). In total, two independent pyrosequencing runs were performed (14 samples with specific MID were pooled in each).

### Data analysis

Reads of individual samples were demultiplexed, sequencing errors were corrected and haplotypes inferred using the program diri_sampler from the ShoRAH software [27]. Error correction included mismatches as well as insertions and deletions. Subsequently, haplotypes were aligned to the 1b HCV reference sequence (GenBank:AJ406073) and translated into amino acid sequences by MEGA (*Molecular Evolutionary Genetics Analysis)*, version 5.0 [28]. Phylogenetic trees were constructed according to the Maximum Likelihood method based on the Tamura-Nei model [29] using MEGA 5.0. Genetic diversity parameters were assessed in HVR1 populations of frequency ≥1%, 2% and 5% by DNA SP version 5 [30]. Such cut-off approach facilitated interpatient comparison of sequence populations of different coverages. HVR1 complexity was represented by the number of haplotypes above each frequency cut-off. Nucleotide diversity per site and the number of substitutions were assessed using DNA SP version 5 with respect to the reference sequence (GenBank:AJ406073). Genetic distances in HCV HVR1 populations were assessed by MEGA. Shannon entropy was calculated according to the following equation:

$$H\left(f\right)=-\sum_{i=1}^{N}f_{i}\log\left(f_{i}\right)$$

Where:

*N* – number of observations (haplotypes),

*f*_*i*_ - frequency of haplotypes

### Statistical methods

Differences in age, alanine aminotransferase activity, viral load, HVR1 complexity, diversity, number of substitutions within HVR1, Shannon entropy, genetic distance, number of polymorphic amino acid positions and number of inner nodes in phylogenetic trees were compared using Mann–Whitney test, while proportions were compared by Fisher’s exact test.

## Results

### Estimation of pyrosequencing and amplification errors based on cloned HVR1 sequence

**Figure 1:**
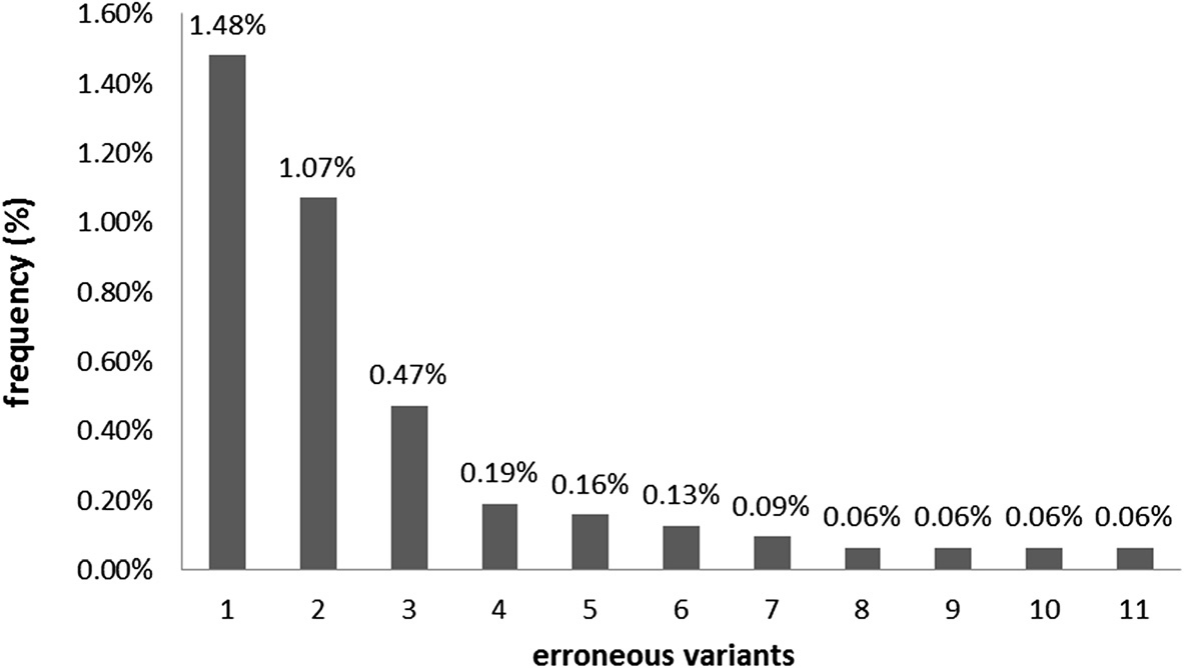
**Frequencies of erroneous variants obtained from sequencing of a single HVR1 clone.** Control experiment performed by sequencing a single HVR1 clone from one pretreatment serum sample presented 11 erroneous variants at frequency between 1.48% and 0.06%. The figure reports, in decreasing order, the frequencies of all 11 variants.

Sequencing of cloned HVR1 fragment provided 3178 reads. After grouping identical reads together, 12 variants were identified (Table 2). Only 96% of reads were identical to the original template. Among 11 erroneous variants, the most abundant constituted 1.48% of all reads, whereas the least abundant was present at a frequency of 0.06% (Figure 1).

**Table 2:** Deep sequencing of cloned HVR1 sample

Number of reads of cloned plasmid (control)	3178
Number of variants	12
Most abundant erroneous variant	1.48%
Least abundant erroneous variant	0.06%
Overall error rate per base	0.05%
Types of errors:	
Insertions	0.04%
Substitutions	0.006%
Deletions	0.002%
Overall insertions at homopolymeric regions	51%
Number of variants after ShoRAH	4
Most abundant erroneous variant after ShoRAH	0.5%
Least abundant erroneous variant after ShoRAH	0.2%

Errors included insertions (83.3%), substitutions (12.5%) and deletions (4.2%). Probability of error occurrence per base was estimated to be 0.04% for insertion, 0.006% for substitution and 0.002% for deletion. Fifty one percent of insertions occurred at homopolymeric regions (four repeats of T). Altogether, the probability of any error per base was 0.05%.

After error correction performed with ShoRAH, four variants were identified: one identical to the template at 99.0% frequency, and three erroneous variants present at frequency of 0.5%, 0.3% and 0.2%, respectively.

### Characteristics of deep sequencing

Over 15 million nucleotides were sequenced (Table 3). After demultiplexing, the median (IQR) of assigned reads was 2540 (2488) per patient sample - 2540 (1790) in responders and 1230 (2816) in non-responders. Following ShoRAH reconstruction, the mean number of haplotypes obtained per patient was 30.6 (38.4 in responders and 23.4 in non-responders). Most abundant haplotype constituted 57.09%, whereas the least abundant only 0.1%. The number of reconstructed haplotypes depends on several factors, including coverage, frequency of the haplotypes and their distance. In order to make a reliable comparison in different patients, we introduced a threshold to the haplotype frequency. The frequency thresholds explored were 1%, 2% and 5%.

**Table 3:** Characteristics of pyrosequencing of pretreatment serum samples from 25 HCV-positive patients receiving PEG-IFN α and ribavirin treatment

Number of sequenced reads aligned to reference genome	72 070
Number of sequenced nucleotides	15 100 000
Median of reads per patient (IQR)	2540 (2488)
Mean number of haplotypes per patient after ShoRAH	30.6
• Responders	38.4
• Non-responders	23.4
Most abundant haplotype	57.09%
Least abundant haplotype	0.1%

### HVR1 genetic heterogeneity

**Figure 2:**
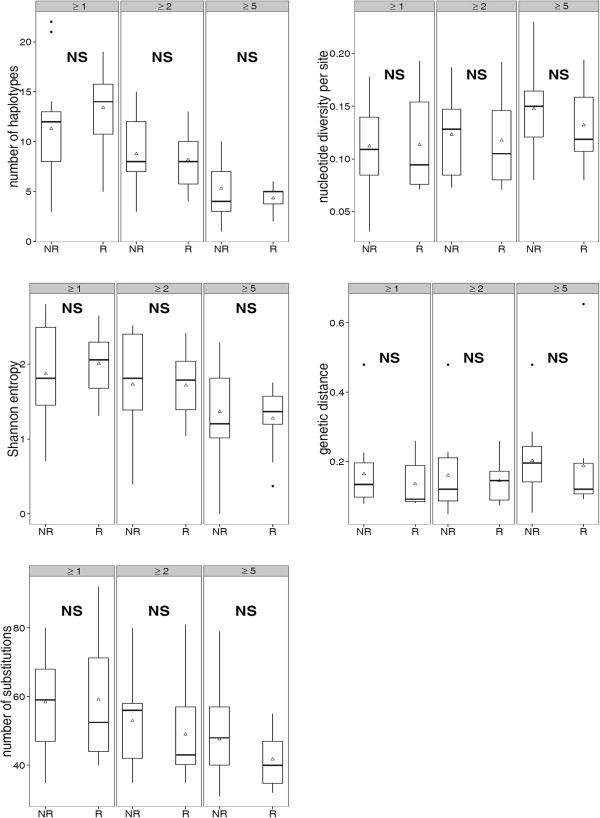
**Heterogeneity parameters of hypervariable region 1 population in responders and non-responders to treatment.** The figure reports the distribution of several parameters describing the heterogeneity of the viral population assessed on hypervariable region 1 by means of massively parallel sequencing and reconstruction of the haplotypes. The results are reported by only considering variants of ≥1%, ≥2% and ≥5% frequency. The horizontal lines, boxes and whiskers indicate the median, IQR (inter-quartile range) and the values within 1.5 × IQR, respectively. Open triangles represent mean values. R- responders, NR – non-responders to treatment, NS-not significant.

HVR1 complexity at ≥5% haplotype frequency cut-off was slightly lower in responders (R) than non-responders (NR); (4.4 *vs* 5.3); (Table 4, Figure 2). Likewise, mean Shannon entropy, mean genetic distance of HVR1 populations and mean number of genetic substitutions and nucleotide diversity per site were also lower in the former group (Table 4, Figure 2). However, these differences did not reach statistical significance. Similarly, when the above analysis was repeated at ≥2% and ≥1% frequency cut-offs, no statistically significant differences were either found.

**Table 4:** HCV HVR1 genetic characteristics in responders and non-responders to PEG-IFN α and ribavirin treatment

	**Responders**	**Non-responders**	***P***
Number of patients	12	13	-
HVR1 complexity (number of haplotypes)			
≥5%	4.4	5.3	NS
≥2%	8.2	8.8	NS
≥1%	13.4	11.3	NS
Mean Shannon entropy			
≥5%	1.28	1.37	NS
≥2%	1.72	1.73	NS
≥1%	2.01	1.87	NS
Mean nucleotide diversity per nucleotide			
≥5%	0.132	0.148	NS
≥2%	0.118	0.123	NS
≥1%	0.114	0.112	NS
Mean genetic distance			
≥5%	0.187	0.203	NS
≥2%	0.145	0.160	NS
≥1%	0.135	0.164	NS
Number of nucleotide substitutions within HVR1			
≥5%	42.8	47.6	NS
≥2%	49.0	52.9	NS
≥1%	59.2	58.4	NS
Percentage of polymorphic amino acid positions			
≥5%	59.3	60.0	NS

### Amino acid variability of HVR1

Within 27 amino acid stretch of HVR1, responders were found to have similar mean number of polymorphic amino acid positions (59.3% ± 9.5%) as non-responders (60% ± 11%); (Table 4). Additional file 1 shows multiple sequence alignment of amino acid sequences of HVR1 populations in responders (R) and non-responders to treatment (NR).

### Phylogenetic analysis

**Figure 3:**
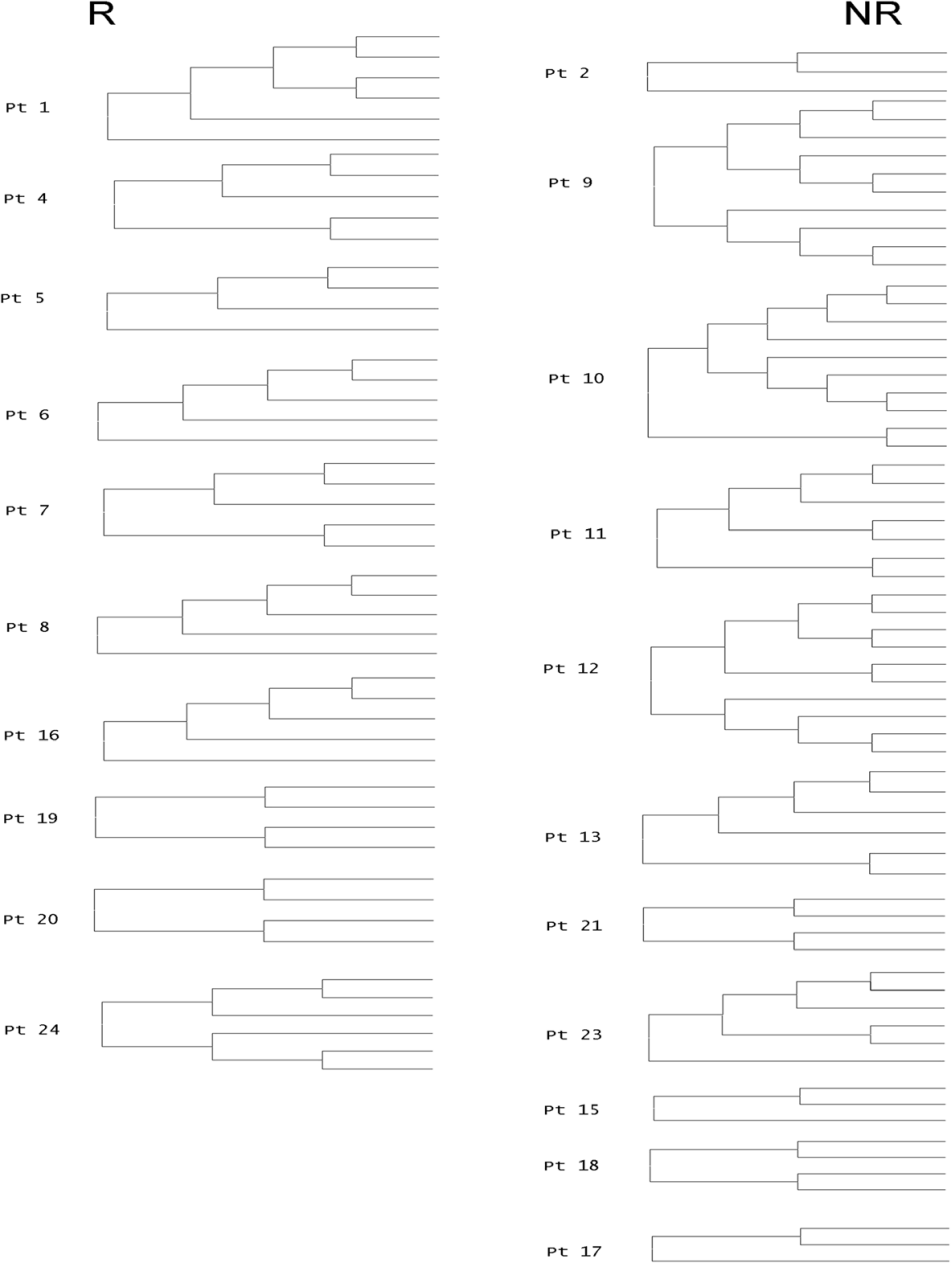
**Phylogenetic analysis of HVR1 populations.** R - responders, NR- non-responders to treatment. Trees were inferred after application of ShoRAH error correction method on haplotypes present at a frequency of ≥5% (for populations constituting at least 3 haplotypes). The evolutionary history was inferred by using the Maximum Likelihood method based on the Tamura-Nei model [29]. Evolutionary analyses were conducted using MEGA 5.0 [28].

Viral populations ≥5% were also analyzed phylogenetically (Figure 3). As shown, populations in non-responders formed more complex patterns of relatedness as manifested by the higher mean number of inner nodes (4.0 ± 2.9 *vs* 2.9 ± 0.7). Nevertheless, this difference was not statistically significant.

## Discussion

A number of previous studies attempted to correlate HVR1 heterogeneity with antiviral treatment outcome, but their results were usually inconclusive and occasionally even contradictory. These discrepancies could be partly due to the use of different techniques: two most commonly used were single strand conformational polymorphism (SSCP) and clonal Sanger sequencing [17-20,31-34]. The latter requires extensive cloning to achieve high sensitivity for minor variants detection, a process that is costly and time-consuming. Thus, studies using this technique rarely included significant number of clones per sample, typically attaining only 15-20% sensitivity. While SSCP has been shown to detect variants constituting as little as 3% of the viral population [10], it is not informative of the nucleotide sequence, the nature of genetic changes or genetic distances between variants. Furthermore, in a mixture of heterogeneous sequences, certain bands may overlap, resulting in underestimation of viral complexity. Our current study, which was based on deep sequencing, overcomes the above shortcomings and represents a novel approach to analysis of HCV heterogeneity.

While our analysis did not find any significant differences in HVR1 heterogeneity between responders and non-responders to antiviral treatment, these results are largely compatible with some previous studies employing SSCP and clonal sequencing. In the study of Pawlotsky et al. [35] based on single strand conformational polymorphism and in the study of Saludes et al. [34] based on clonal sequencing, no significant differences in pretreatment HVR1 complexity were observed between responders and non-responders. Similar results were reported in a study of re-treated patients with advanced fibrosis [33], while Abbate et al. [31] found that low pretreatment HVR1 heterogeneity correlated with early response (EVR), but not with SVR. A number of other studies found no correlation between HVR1 complexity and treatment outcome [17,18,36].

In our study, such HVR1 heterogeneity parameters, as nucleotide diversity per site, genetic distance, and number of nucleotide substitutions also did not differ significantly between responders and non-responders. These findings are similar to several earlier studies [20,34,37,38]. In the only published study using deep sequencing approach, there were no differences in pretreatment complexity parameters (e.g. Shannon entropy) between immediate virological responders and non-responders. However, the final treatment outcome was not reported [25].

Lack of statistically significant differences in analyzed heterogeneity parameters between responders and non-responders suggest that the heterogeneity generated by minor variants detectable by deep sequencing has no effect on treatment outcome. Alternatively, it may be speculated that the analyzed depth of frequency is still insufficient to detect minor variants whose heterogeneity would have clinical significance.

Some recent studies brought attention to the problem of inherent ultra-deep sequencing errors affecting the detection of minor variants of the quasispecies population [26,39,40]. In our analysis, the internal control experiment using cloned HVR1 revealed the overall sequencing error to be 0.05% per nucleotide, comprising mostly of insertions and occurring predominantly in homopolymeric regions. This error rate contributed to the high proportion of erroneous sequences (4% of total reads, the most abundant erroneous variant being present at a frequency of 1.48%). To minimize the risk of including erroneous variants into analysis, we implemented *Sho*RAH error correction method, which allowed for correction of 99% of reads reducing both the absolute number and frequency of erroneous variants. Thus, error correction methods should be used to facilitate analysis of minor quasispecies by pyrosequencing.

## Conclusions

There were no significant differences in the pretreatment HVR1 heterogeneity parameters such as complexity, Shannon entropy, nucleotide diversity per site, genetic distance and the number of genetic substitutions between responders and non-responders. Thus, pretreatment HVR1 quasispecies composition and heterogeneity analysis seems to have limited value for the prediction of treatment outcome.

## Competing interests

The authors declare that they have no competing interest.

## Authors’ contributions

KCC participated in the design of the study and its coordination, amplified HVR1 sequences, carried out the molecular genetic studies deep sequencing of HVR, calculated genetic heterogeneity parameters, performed the statistical analysis and drafted the manuscript. OZ made reconstruction of HVR1 populations and correction of sequencing error, calculated genetic heterogeneity parameters, prepared figures and helped to draft the manuscript. KP prepared HVR1 amplicons for sequencing, made sequence alignments and calculated genetic heterogeneity parameters. TL participated in the design of the study and helped to draft the manuscript. KM calculated genetic heterogeneity parameters. IBO prepared the database of results and tables. AP made RNA isolation from samples, collected and analyzed clinical and virological data of patients. RP participated in the design of the study, interpretation of results and helped to draft the manuscript. HB participated in the design of the study, treated patients and provided information about the study, obtained informed consents and collected clinical and virological data of patients. AH participated in the design of the study, provided information about the study to the patients, collected clinical and virological data of patients, obtained informed consents. MR participated in the design of the study and helped to draft the manuscript. All authors read and approved the final manuscript.

## Pre-publication history

The pre-publication history for this paper can be accessed here:

http://www.biomedcentral.com/1471-2334/14/389/prepub

## Supplementary Material

Additional file 1 Multiple sequence alignment of amino acid sequences of HVR1 populations in responders (R) and non-responders to treatment (NR). Click here for file
