# Supplementary material for: Deep sequencing of hepatitis C virus hypervariable region 1 reveals no correlation between genetic heterogeneity and antiviral treatment outcome
Source: BMC Infect Dis. 2014 Jul 13;14:389. doi: 10.1186/1471-2334-14-389 (PMC4226954; doi:10.1186/1471-2334-14-389)
Supplement: Additional file 1 — Multiple sequence alignment of amino acid sequences of HVR1 populations in responders (R) and non-responders to treatment (NR). [file 1471-2334-14-389-S1.pdf]

Multiple sequence alignment of amino acid sequences of HVR1 populations

in responders (R) and non-responders to treatment (NR)*

R

```
                                                                HVR1
Pt_1

#AJ406073                KVLIVMLLFA GVDGGHTHVT GGAAAHAAYG FRHLFSPGSAQ KLQLV
#hap_16_freq_27.3        .......... .......R.. ..T..YTTS. LTS..T..PS. RI..I
#hap_20_freq_21.143174   .......... .......R.. ..T..YTTSR LTS..T..PS. RI..I
#hap_5_freq_11.183094    .......... .......R.. ..T..YTTSR LTS..T..PS. RI..I
#hap_3_freq_8.623567     .......... .......... ..TTG.TTSR .VSM..S.PS. .I..M
#hap_8_freq_7.360572     .......... .......R.. ..T..YTTS. LTS..T..PS. RI..I
#hap_12_freq_6.162473    .......... .......R.. ..T..YTTS. LTS..T..PS. QI..I

Pt_3

#AJ406073                KVLIVMLLFA GVDGGHTHVT GGAAAHAAYG FRHLFSPGSAQ KLQLV
#hap_16_freq_39.428549   .......... ....ANIY.. ..Q.SRTTR. .AG...A.... .I..I
#hap_20_freq_34.971555   .......... .....Q.Y.. ..Q.SRTTR. .AG...A.... .I..I

Pt_4

#AJ406073                KVLIVMLLFA GVDGGHTHVT GGAAAHAAYG FRHLFSPGSAQ KLQLV
#hap_12_freq_21.191948   .......... .....Q.LL. ..Q..RTTQ. LA...N..PS. .I...
#hap_37_freq_15.720680   .......... .....Q.L.. ..Q..RTTQ. LA...N..PS. .I...
#hap_34_freq_7.186187    .......... ....VP.... ..Q..RTTQ. .AS..IS.PS. .I...
#hap_19_freq_5.123561    .......... ....AP.... ..Q..RTTQ. .AS..IS.PS. .I...
#hap_31_freq_5.063236    .......... ....A..... ..Q.SRTTQ. LAS..IS.PS. .I...

Pt_5

#AJ406073                KVLIVMLLFA GVDGGHTHVT GGAAAHAAYG FRHLFSPGSAQ KLQLV
#hap_11_freq_36.258013   .......... .........S ..S..R.TH. .TS..T..PS. .I..I
#hap_1_freq_30.544806    .......... .....N...S ..S..R.TH. .TS..T..PS. .I..I
#hap_18_freq_11.023923   .......... .....N.R.. ..SV.QTTR. LTS..N..PS. .I..I
#hap_14_freq_5.817838    .......... .....N...S ..S..R.TH. .TS..T..PS. .I..I

Pt_6

#AJ406073                KVLIVMLLFA GVDGGHTHVT GGAAAHAAYG FRHLFSPGSAQ KLQLV
#hap_1_freq_20.608035    .......... .....G..T. ....G.TV.. .AG..A..PS. .I..I
#hap_29_freq_18.821522   .......... .....D..T. ....G.TV.. .AG..A..PS. .I..I
#hap_4_freq_12.638315    .......... .....D..T. ....G.TVH. .AG..A..PS. .I..I
#hap_10_freq_10.529713   .......... .....G..T. ....G.TVH. .AG..A..PS. .I..I
#hap_35_freq_10.042218   .......... ........T. ..KVSRDTSA LTS.....AS. .I..I
```

* Top sequence corresponds to reference genotype 1b sequence (GenBank: AJ406073). HVR1 is marked by brackets. Dots indicate consensus positions, dashes indicate gaps in alignments while asterisks indicate stop codons. Shown are sequences representing ≥5% of frequency.

Pt_7

```
#AJ406073               KVLIVMLLFA GVDGGHTHVT GGAAAHAAYG FRHLFSPGSAQ KLQLV
#hap_12_freq_34.335146  .......... .....P...A ..T..RTTQ. LTS...V.PS. .I...
#hap_11_freq_12.643542  .......... .....P.... ..T..RTTQ. .TS...A.PS. .I...
#hap_15_freq_10.036378  .......... .....P...A ..T..RTTQ. LTS...A.PS. .I...
#hap_5_freq_7.271620    .......... .....P...A ..T..RTTQ. LTS..TS.PS. .I...
#hap_8__freq_6.288163   .......... .....P...A ..T..RTTQ. LTS..KR.PS. .I...
```

Pt_8

```
#AJ406073               KVLIVMLLFA GVDGGHTHVT GGAAAHAAYG FRHLFSPGSA QKLQLV
#hap_48_freq_17.521384  .......... .....D.... ..T..YT.R. L.P..A..PK .NI..I
#hap_17_freq_16.645096  .......... .......YA. ....GRTTF. LAS.LA..PS ..I..I
#hap_42_freq_13.602756  .......... .....D.... .ET.GR..AR .SVF.TA.PS ..I..I
#hap_2_freq_11.499667   .......... .....D.... ..T..YT.R. LTS..TR.PK .NI..I
#hap_26_freq_8.974066   .......... .....D.... ..T..YT.R. .TS..TR.PK .NI..I
```

Pt_16

```
#AJ406073               KVLIVMLLFA GVDGGHTHVT GGAAAHAAYG FRHLFSPGSA QKLQLV
#hap_13_freq_44.928136  .......... .......QTM ...V..TTQ. LTS..LR.PN ..I..I
#hap_5_freq_9.660595    .......... .......QTM ...V..TTQ. LTS..LR.PN ..I..I
#hap_10_freq_8.999881   .......... .......QTM ...V..TTQ. LTS..LR.PN ..I..I
#hap_12_freq_7.673817   .......... .....-.QTM .....YNTE. LTS..VQ.PN .NI..I
#hap_7_freq_6.552028    .......... ....A.PNAM ...V..TTQ. LTS..LR.PN ..I..I
```

Pt_19

```
#AJ406073               KVLIVMLLFA GVDGGHTHVT GGAAAHAAYG FRHLFSPGSA QKLQLV
#hap_14_freq_23.179557  .......... .....-.Y.. ........RS .AGF.TA.PS ..I...
#hap_17_freq_12.288348  .......... .....-.Y.. ........RS .AGF.TA.PS ..I...
#hap_7_freq_10.803667   .......... .....DS..I ...E.R..*. L.G..TR..Y ..I...
#hap_12_freq_5.501986   .......... .....P.Y.I ..SV..G.R. LSS..TA..Q ..I...
```

Pt_20

```
#AJ406073               KVLIVMLLFA GVDGGHTHVT GGAAAHAAYG FRHLFSPGSA QKLQLV
#hap_4_freq_35.218426   .......... S...K.LY.. ..SV.RSVSK .TS..TQ.PS .HI...
#hap_1_freq_25.397660   .......... .....-.Y.. ........RS .AGF.TA.PS ..I...
#hap_3_freq_19.676378   .......... .....-.Y.. ........RS .AGF.TA.PS ..I...
#hap_5_freq_16.080856   .......... S...K.LY.. ..SV.RSVSK .TS..TQ.PS .HI...
```

Pt_24

```
#AJ406073               KVLIVMLLFA GVDGGHTHVT GGAAAHAAYG FRHLFSPGSA QKLQLV
#hap_60_freq_12.481027  .......... ....R..D.. ..E.GRTTR. L.R..T..PS .SI...
#hap_38_freq_9.702394   .......... .....-.Q.. ..K.GRTTS. .GR...H.PS .SI...
#hap_13_freq_7.441796   .......... .....Q.K.. ..Q.GRTTS. LGR..TA.PS .SI...
#hap_19_freq_6.710272   .......... ....R..D.. ..E.GRTTS. .GR...H.PS .SI...
#hap_20_freq_6.697649   .......... .....-.Q.. ..E.GRTTS. .GR...H.PS .SI...
#hap_90_freq_5.576174   .......... ....R..D.. ..E.GRTTR. .TG..A..PS .SI...
```

Pt_25

```
#AJ406073               KVLIVMLLFA GVDGGHTHVT GGAAAHAAYG FRHLFSPGSA QKLQLV
#hap_35_freq_45.231685  .......... .....-.Y.. .....Q..RS .AGF.TA.PS ..I...
#hap_21_freq_6.336005   .......... .....-.Y.. .....QG.RS .AGF.TA.PS ..I...
```

NR

```
Pt_2

#AJ406073                KVLIVMLLFA GVDGGHTHVT GGAAAHAAYG FRHLFSPGSAQ KLQLV
#hap_2_freq_52.305680    .......... .....G..TV ...Q.RTTS. .AS..KL.PS. ....I
#hap_1_freq_24.665732    .......... .....G..TV ...Q.R.TS. .AS..KL.PS. ....I
#hap_3_freq_23.028589    .......... .....G..TV ...Q.RTTS. .AS..KL.PS. .....

Pt_9

#AJ406073                KVLIVMLLFA GVDGGHTHVT GGAAAHAAYG FRHLFSPGSAQ KLQLV
#hap_8_freq_11.354688    .......... .....E..T. .....RTTH. LAS..T..AQ. .I..I
#hap_2_freq_11.227527    .......... .....DPYA. .....RTTH. LAS..T..AQ. .I..I
#hap_9_freq_10.576891    .......... .....DPYA. .....RTTH. LAS..T..AQ. .I..I
#hap_3_freq_9.481884     .......... .....E..T. .....RTTH. LAS..T..AQ. .I..I
#hap_6_freq_9.243186     .......... .....DPYA. .....RTTH. LAS..T..AQ. .I..I
#hap_4_freq_8.754121     .......... .....D..T. .....RTTQ. LAS..T..AQ. .I..I
#hap_11_freq_8.102100    .......... .....E..T. .....RTTH. LAS..T..AQ. .I..I
#hap_5_freq_7.547972     .......... .....DPYA. .....RTTH. LAS..T..AQ. .I..I
#hap_10_freq_7.515144    .......... .....G..T. .....RTTH. LAS..T..AQ. .I..I
#hap_13_freq_6.326991    .......... .....G..T. .....RTTQ. LAS..T..AQ. .I..I

Pt_10

#AJ406073                KVLIVMLLFA GVDGGHTHVT GGAAAHAAYG FRHLFSPGSAQ KLQLV
#hap_1_freq_14.312893    ...V...... .....L.QTI .R...RS.SV .TS..KC.... .I..I
#hap_7_freq_9.983245     ...V...... .....P.QTN .R...QS.SV .TS..*C.... .I..I
#hap_6_freq_9.610717     ...V...... .....P.QTI .R...RS.SV .TS..*C.... .I..I
#hap_13_freq_9.356483    ...V...... .....P.QTN .R...QS.SV .TS..*V.... .I..I
#hap_8_freq_9.073097     ...V...... .....P.QTN .RT..QS.SV .TS..*C.... .I..I
#hap_10_freq_9.053612    ...V...... .....P.QTI .RT..RS.SV .TS..*C.... .I..I
#hap_1_freq_8.849703     ...V...... .....P.QTN .R...QS.SV .TS..*C.... .I..I
#hap_11_freq_7.603696    ...V...... .....P.QTM .....V*TSV LTS..*..... .I..I
#hap_2_freq_7.396565     ...V...... .....P.QTI .R...RS.SV .TS..KR.... .I..I
#hap_3_freq_5.092657     ...V...... .....P.QTM .....VR.SV LTS..*..... .I..I

Pt_11

#AJ406073                KVLIVMLLFA GVDGGHTHVT GGAAAHAAYG FRHLFSPGSAQ KLQLV
#hap_3_freq_28.926727    .......... .......QTV ....GYT.S. LTSM..A.PS. NI...
#hap_6_freq_22.474484    .......... .......QTV ....GYT.S. LTSIL.R.PS. NI...
#hap_7_freq_11.623517    .......... .....YLYA. ...V.RTTS. LTG..T..PS. .I...
#hap_2_freq_11.377759    .......... ......LYA. ...V.RTTS. LTG..T..PS. .I...
#hap_1_freq_11.369550    .......... .......YT. ...V.RTTS. LTG..*..PS. .I...
#hap_4_freq_8.773702     .......... .......YT. ...V.RTTS. LTG..*..PS. .I...
#hap_5_freq_5.454261     .......... .....YLYA. ...V.RTTS. LTG..T..PS. .I...

Pt_12

#AJ406073                KVLIVMLLFA GVDGGHTHVT GGAAAHAAYG FRHLFSPGSAQ KLQLV
#hap_7_freq_14.761413    .......... .......QTV ....GYT.S. LTSIL.R.PS. NI...
#hap_11_freq_13.974557   .......... .......QTV ....GYT.S. LTSM..A.PS. NI...
#hap_3_freq_13.295148    .......... .....YLYA. ...V.RTTS. LTG..T..PS. .I...
#hap_1_freq_9.225205     .......... .......YT. ...V.RTTS. LTG..*..PS. .I...
#hap_8_freq_7.216281     .......... .......QTV ....GYT.S. LTSIL.R.PS. NI...
#hap_10_freq_7.054568    .....L.... .....Y.Q.. ..R.GQTTH. .AS..*T.PS. .I..M
#hap_12_freq_7.015768    .......... ....--.Y.. ..R.G.TNS. .TS..AS.PT. .I...
#hap_2_freq_6.966658     .....L.... .......Q.. ..R.GQTTH. YWS..*A.PS. .I..M
#hap_9_freq_6.192419     .......... .......K.. ..T...TTH. LAS..*T.PS. .I..M
#hap_5_freq_5.838605     .......... .......QTV ....GYT.S. LTSM..A.PS. NI...
```

```
Pt_13

#AJ406073               KVLIVMLLFA GVDGGHTHVT GGAAAHAAYG FRHLFSPGSAQ KLQLV
#hap_1_freq_8.133845    .......... .....R.R.S ..T...TTS. L.V.....P.. .I...
#hap_12_freq_8.402748   .......... .....V.R.S ..T...TTS. .VS.....P.. .I...
#hap_13_freq_12.311684  .......... .....Q...I ..S..FTTS. .AG.LTR.P.. .I...
#hap_17_freq_9.143148   .......... .....Y...I ..SL.RS.F. LTGFL.Q.PS. .....
#hap_18_freq_6.354064   .......... .....-.Y.. ....GRT.F. .AS.LN..P.. .....
#hap_21_freq_13.004474  .......... .....Q.R.S ..T..RNTF. LVSFL...P.. .I...

Pt_14

#AJ406073               KVLIVMLLFA GVDGGHTHVT GGAAAHAAYG FRHLFSPGSAQ KLQLV
#hap_2_freq_84.402961   .......... ....V..E.. ..T..RTTS. LTS..IS.PS. NI..I

Pt_15

#AJ406073               KVLIVMLLFA GVDGGHTHVT GGAAAHAAYG FRHLFSPGSA QKLQLV
#hap_28_freq_41.569919  .......... .....P.R.. ..TE....SS LVRF.T..P. .....I
#hap_10_freq_27.762601  .......... .....P.R.. ..TE....SS LVRF.T..P. .....I
#hap_19_freq_6.710451   .......... .....P.R.. ..TE....SS LVRF.T..P. .....I

Pt_17

#AJ406073               KVLIVMLLFA GVDGGHTHVT GGAAAHAAYG FRHLFSPGSA QKLQLV
#hap_34_freq_24.680097  .......... .....-.Y.. ..S....PV. L.G..TV.AN .RI...
#hap_8_freq_11.193294   .......... .......R.A ..V.GQTTK. .TS..*.... .RI...
#hap_20_freq_10.364548  .......... .....-.... ..T....PST .TR.....PS .RI...

Pt_18

#AJ406073               KVLIVMLLFA GVDGGHTHVT GGAAAHAAYG FRHLFSPGSA QKLQLV
#hap_14_freq_57.097667  .........S ....R.L.A. ..T.GRTTH. LTS...L.AS ..I..I
#hap_7_freq_15.218811   .........S ....R.L.A. ..T.GRTTH. LTS...L.AS ..I..I
#hap_1_freq_11.151133   .........S ....R.L.A. ..T.G.TTR. LTS...L.AS ..I..I
#hap_12_freq_10.348341  .........S ....R.L.A. ..T.G.TTR. LTS...L.AS ..I..I

Pt_21

#AJ406073               KVLIVMLLFA GVDGGHTHVT GGAAAHAAYG FRHLFSPGSA QKLQLV
#hap_23_freq_35.986546  .......... ....CQ..T. .....YTTSS IVS..R..PS ..I...
#hap_4_freq_17.383868   .......... ....CQ.YT. ..SV.RG.S. ITS.....PS ..I...
#hap_30_freq_14.065090  .......... ....*Q...A ..S..RDTS. ITG...L.AS ..I...
#hap_27_freq_5.477279   .......... ....CQ..T. .....YTTSS IVS..R..PS ..I...

Pt_22

#AJ406073               KVLIVMLLFA GVDGGHTHVT GGAAAHAAYG FRHLFSPGSA QKLQLV
#hap_37_freq_42.290895  .......... .....Q.R.. ..SS.F.TRS .VS...S.PS ..I...
#hap_25_freq_23.425837  .......... .....Q.R.. ..SS.FTTS. LVS...S.PS ..I...

Pt_23

#AJ406073               KVLIVMLLFA GVDGGHTHVT GGAAAHAAYG FRHLFSPGSA QKLQLV
#hap_30_freq_32.618488  .......... ....A..Y.. ..L.SQ.TH. ITS...F.PN ......
#hap_8_freq_10.459786   .......... ....A..Y.. ..Q.SKT... ITS...F.PN ......
#hap_28_freq_5.936241   .......... ....TG.R.. ..T..QTTR. .AS..T..P. .....I
#hap_14_freq_5.919826   .......... ....AQ.Y.. ..L.SQ.TH. ITS...F.PN ......
#hap_26_freq_5.783969   .......... ....A..Y.. ..H.SQ.TH. ITS...F.PN ......
#hap_50_freq_5.460061   .......... ....AD.Y.. ..Q.SKT... ITS...F.PN ......
```
